# The non-invasive serum biomarkers contributes to indicate liver fibrosis staging and evaluate the progress of chronic hepatitis B

**DOI:** 10.1186/s12879-024-09465-z

**Published:** 2024-06-26

**Authors:** Shaoxiong Zeng, Zhenzhen Liu, Bilun Ke, Yiwang Zhang, Qian Wang, Siwei Tan

**Affiliations:** 1 Department of Gastroenterology, The Third Affiliated Hospital of Sun Yat-Sen University, 600 Tianhe Road, Guangzhou, Guangdong Province 510630 China; 2 Department of Gastroenterology, Huazhong University of Science and Technology Union Shenzhen Hospital (Nanshan Hospital), Shenzhen, Guangdong Province 518052 China; 3 Department of Radiology, The Third Affiliated Hospital of Sun Yat-Sen University, Guangzhou, Guangdong Province 510630 China; 4 Department of Pathology, The Third Affiliated Hospital of Sun Yat-Sen University, Guangzhou, Guangdong Province 510630 China

**Keywords:** Chronic hepatitis B, Liver fibrosis, Liver cirrhosis, Liver biopsy, Non-invasive serum biomarkers

## Abstract

**Background:**

This study aimed to evaluate the diagnostic abilities of the non-invasive serum biomarkers to predict liver fibrosis staging and evaluate the progress of hepatitis B.

**Methods:**

We enrolled 433 patients with chronic HBV infection had complete medical data available for the study, who underwent percutaneous liver biopsy. The extent of fibrosis was assessed using the modified METAVIR score. The predictive values of the non-invasive serum biomarkers were evaluated by the areas under the receiving operator characteristics curves (AUROCs) with 95% confidence intervals.

**Results:**

The proportion of males with progressive stages of liver fibrosis was relatively larger, and the average age of patients with cirrhosis stages is older than the non-cirrhotic stages. We found PLT, GGT, ALP, TB, FIB4 and GPR to be significantly associated with liver fibrosis in our cohort. GGT showed a sensitivity of 71.4% and specificity of 76.7% in distinguishing cirrhosis (F4) from non-cirrhotic stages (F1-3), with an AUROC of 0.775 (95%CI 0.711–0.840).The AUROCs of the GPR in distinguishing cirrhosis (F4) from non-cirrhotic stages (F1-3) was 0.794 (95%CI 0.734–0.853), but it had a lower sensitivity of 59.2%. Additionally, GGT, FIB4, and GPR could differentiate advanced fibrosis (F3-4) from non-advanced fibrosis (F1-2) among individuals with chronic hepatitis B, with AUROCs of 0.723 (95%CI 0.668–0.777), 0.729 (95%CI 0.675–0.782), and 0.760 (95%CI: 0.709–0.811) respectively.

**Conclusions:**

GGT was a better biomarker to distinguish cirrhosis (F4) from non-cirrhotic stages (F1-3), while GPR was a better biomarker to identify advanced fibrosis (F3-4) and non-advanced fibrosis (F1-2) in patients with chronic hepatitis B.

**Supplementary Information:**

The online version contains supplementary material available at 10.1186/s12879-024-09465-z.

## Background

Chronic hepatitis B virus infection is a global public health threat that causes considerable liver-related morbidity and mortality. The worldwide estimated prevalence of chronic HBV infection in 2016 was 3.5% with 257 million people living with chronic infection [1]. HBV infection significantly increases the risk of liver fibrosis, cirrhosis, and hepatocellular carcinoma (HCC) [2]. Early and accurate assessment of the degree of liver fibrosis in patients with HBV infection can not only guide the timing of antiviral therapy, but also predict the long-term clinical prognosis of HBV infection [3].

Liver biopsy is the gold standard for determining fibrosis stage and for stratifying risk of hepatocellular carcinoma (HCC) [4]. However, liver biopsy is an invasive technique, which may cause complications such as bleeding and threaten the safety of patients, thus limiting its widespread application in clinical practice. The diagnostic accuracy of this technique is also decreased by intra and interobserver variability in pathological assessment [5]. In addition, the biopsy sample size obtained by puncture may not be sufficient for accurate staging of liver fibrosis, and the accuracy of liver biopsy may be affected by inaccurate sampling sites [6]. Therefore, non-invasive methods to detect serum biomarkers and corresponding indicators have been used to assess liver fibrosis stage. Such as fibrosis index based on four factors (FIB-4), aspartate aminotransferase (AST) to platelet ratio index (APRI), gamma-glutamyl transpeptidase (GGT) to platelet ratio (GPR), aspartate aminotransferase (AST) to alanine aminotransferase (ALT) ratio (AAR), Albumin-bilirubin (ALBI) score, etc [7–9]. APRI and FIB-4 can be used to stage liver fibrosis by routine blood tests of AST, alanine aminotransferase (ALT), and platelet count (Plt), and have been recommended by WHO guidelines and many other guidelines for the assessment of liver fibrosis stage in resource-limited countries [10–12].

At present, there is a lack of research on the predictive ability of the above non-invasive markers for the staging of hepatitis B liver fibrosis. Therefore, this study mainly explored the diagnostic ability of the above non-invasive biomarkers for liver fibrosis staging of hepatitis B.

## Materials and methods

### Patients tissue samples

This study was performed in a retrospective manner. A total of 433 patients with chronic HBV infection (CHB), who underwent percutaneous liver biopsy examinations in a clinical setting between 2017-01-01 and 2022-01-01, were enrolled from the department of gastroenterology of the Third Affiliated Hospital of Sun Yat-Sen University.

All 433 patients had complete medical data available for the study. Patients were diagnosed as CHB according to AsianPacific clinical practice guidelines about CHB patients’ management [2]. HBV Markers (HBsAg, anti-HBs, HBeAg, anti-HBe and anti-HBc) were detected by an i2000 immunoassay instrument (Abbott Laboratories).

All CHB patients were already starting regular followed up visits in the hospital and underwent laboratory investigations and diagnostic liver biopsy for identification of their fibrosis stage before starting or declining antiviral treatment. History or evidence of the following conditions resulted in exclusion during patient recruitment: human immunodeficiency virus infection, other hepatitis infections, and any form of cancer or liver disease, such as non-alcoholic fatty liver disease or alcoholic liver disease.

All tissues and medical data were collected with the patients’ and volunteers’ informed consent prior to inclusion in the study, and this study protocol was approved by the Institute Research Ethics Committee of the Third Affiliated Hospitals of Sun Yat-Sen University.

### Histopathological analysis

The extent of fibrosis was assessed using the modified METAVIR score as follows: F0, no fibrosis; F1, portal fibrosis without septa; F2, portal fibrosis and a few septa; F3, numerous septa without cirrhosis; and F4, cirrhosis [13]. The METAVIR grading system was used to assess hepatic inflammatory activity [14]. Staging fibrosis and grading activity were undertaken by an experienced pathologist who specialized in liver pathology.

### Clinical and laboratory data collection

Electronic medical charts were retrospectively reviewed. The following data were collected: age, sex, clinical presentation, alanine aminotransferase (ALT), aspartate aminotransferase (AST), gamma-glutamine transferase (GGT), alkaline phosphatase (ALP), total bilirubin (TB), albumin (ALB), platelets count (PLT). FIB-4 was calculated as follows [15]: FIB4 = [age (years)×AST (IU/L)]/[PLT (10^9^/L)×ALT ^1/2^ (IU/L)]; APRI was calculated as follows [16]: APRI = (AST/upper limit of normal)/PLT (10^9^/L)×100, the AST upper limit was 40 IU/L and ranged from 7 to 40 IU/L; ALBI score was calculated as follows [17]: Log10 TB (µ mol/L)×0.66 + ALB (g/L)×(-0.085); AAR was calculated as follows [18]: AAR = AST (IU/L)/ALT (IU/L); GPR was calculated as follows [18]: GPR = GGT (IU/L)/PLT (10^9^/L).

### Statistical analyses

Statistical analyses were performed using SPSS version 18.0 (SPSS Inc., Chicago, IL, USA). Categorical and continuous variables were reported as the frequency and mean ± standard errors, respectively. Comparative analyses of more than two groups were performed using analysis of variance (ANOVA). Significant differences were assessed by the chi-squared test and Fisher’s exact test for categorical variables. The correlations of differences were considered statistically significant at *p* < 0.05. The diagnostic performances of the biomarkers were assessed by receiver operating characteristic (ROC) curves. The areas under the ROC curves (AUCs) were calculated with 95% confidence intervals. The cut-off values were chosen at maximizing the sensitivity and specificity and diagnostic accuracy. A two-tailed *p* value < 0.05 was considered statistically significant.

## Results

### Baseline patient characteristics

A total of 433 patients with hepatitis B virus (HBV) infection, comprising 331 (76.44%) men and 102 (23.56%) women, with a mean age of 39.40 ± 9.987 years, were enrolled in this study. Based on the liver biopsy examinations, the number of patients in the F0, F1, F2, F3 and F4 stage was 93 (21.48%), 83 (19.17%), 111 (25.64%), 75 (17.31%) and 71 (16.40%), respectively, according to the METAVIR score (Table 1). One-way ANOVA test demonstrated statistically differences in age distribution among the 5 groups, and the average age of patients in F4 group is older than the other groups by the S-N-K analysis, while there is no difference between the other groups. In further analysis, chi-square tests were used to observe gender distribution among the 5 groups. There is no statistical difference in the gender composition ratio (male/female) between the 5 groups. However, the gender composition ratio of F3 and F4 group was higher than that of F0, F1 and F2 group (5.636 vs. 2.588, 𝒳^2^ = 8.813, *p* = 0.003), which predicted a relatively larger proportion of males with progressive stages of liver fibrosis.

**Table 1:** Baseline demographic characteristics of the 433 patients with Hepatitis B virus infection at the time of liver biopsy

Fibrosis staging	Total	F0	F1	F2	F3	F4	*P*
Patient number(%)	433	93(21.48)	83(19.17)	111(25.64)	75(17.31)	71(16.40)	
Age, years	39.40 ± 9.987	37.13 ± 8.293	37.83 ± 8.564	37.17 ± 10.250	39.65 ± 8.014	47.41 ± 11.114	< 0.001*
Gender, female/male	102/331	27/66	26/57	27/84	12/63	10/61	NS^&^

*The average age of patients in F4 group is older than the other groups by the S-N-K analysis;

NS, no significant difference; ^&^The chi-square test was performed after F0-F2 was combined into one group and F3-F4 was combined into another group, the gender composition ratio of F3 and F4 group was higher than that of F0, F1 and F2 group (5.636 vs. 2.588, 𝒳^2^ = 8.813, *p* = 0.003)

### Comparison of the laboratory characteristics in each stage of fibrosis

**Fig. 1:**
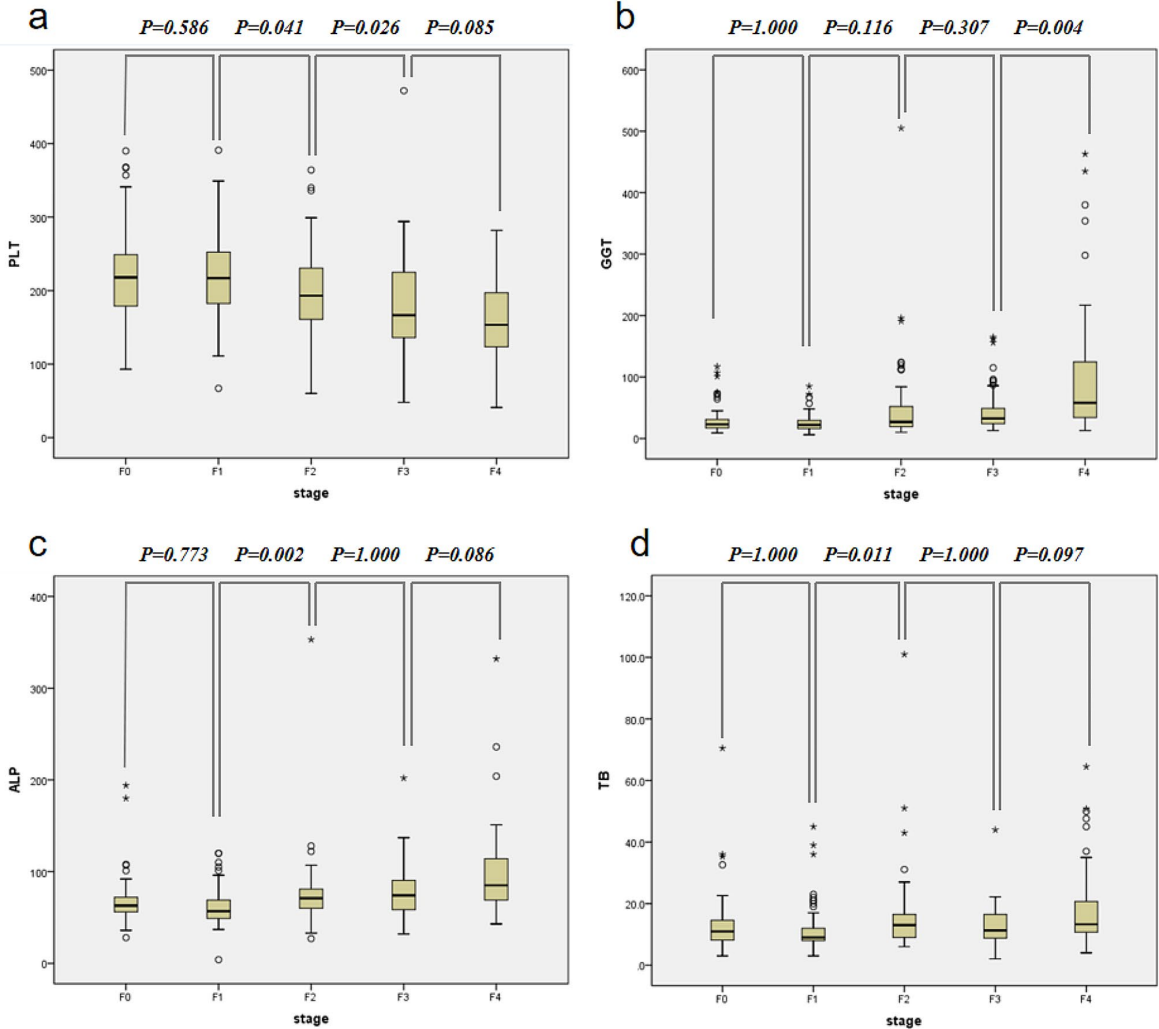
The non-invasive serum biomarkers in each fibrosis stage. The diagnostic ability of the non-invasive serum biomarkers in differentiating liver fibrosis stage were evaluated. **(a)** PLT was able to distinguish stage 2 from 1 and stage 3 from 2(*P* < 0.05). **(b)** GGT was able to differentiate stage 4 from 3 (*P* < 0.05). **(c) **ALP was able to differentiate stage 2 from 1(*P* < 0.05). **(d)** TB was able to differentiate stage 2 from 1(*P* < 0.05). *P*-values < 0.05 were considered statistically significant

The mean ALT, AST, GGT, ALP, TB and PLT were demonstrated in Table 2. And Five of the six serum markers apart from ALT presented statistical differences among individuals with different liver fibrosis stages. In fully adjusted models, we found 4 (PLT, GGT, ALP and TB) of the 6 serum markers to be significantly associated with liver fibrosis (Fig. 1): PLT showed statistical differences between F1 and F2 group (*p* = 0.041) or F2 and F3 group (*p* = 0.026), respectively; GGT showed statistical differences between F3 and F4 group (*p* = 0.004); ALP showed statistical differences between F1 and F2 group (*p* = 0.002); TB showed statistical differences between F1 and F2 group (*p* = 0.011).

**Table 2:** Comparison of the laboratory characteristics of the 433 patients in each stage of fibrosis

Fibrosis staging	F0	F1	F2	F3	F4	*P*
Patient number	93	83	111	75	71	
ALT(U/L)	30(27)	30(21)	37(30)	36(39)	35(58)	0.181
AST(U/L)	23(10)	26(12)	30(14)	32(23)	36.5(32)	< 0.001
GGT(U/L)	23(14)	22(14)	27(34)	32.5(25)	58(92)	< 0.001
ALP(U/L)	63(17)	57(21)	71(22)	74(33)	85(45)	< 0.001
TB(umol/L)	11(6.5)	9(5)	13(7.8)	11.3(8.0)	13.25(10.3)	< 0.001
PLT(10E^9^/L)	221.22 ± 60.20	217.92 ± 57.67	197.00 ± 58.65	178.28 ± 68.01	157.98 ± 61.60	< 0.001

Only PLT follows normality presented as the mean ± standard deviation. Other continuous variables are presented as the median (interquartile range);

### Comparison of the FIB4, APRI, ALBI, AAR and GPR in each stage of fibrosis

**Fig. 2:**
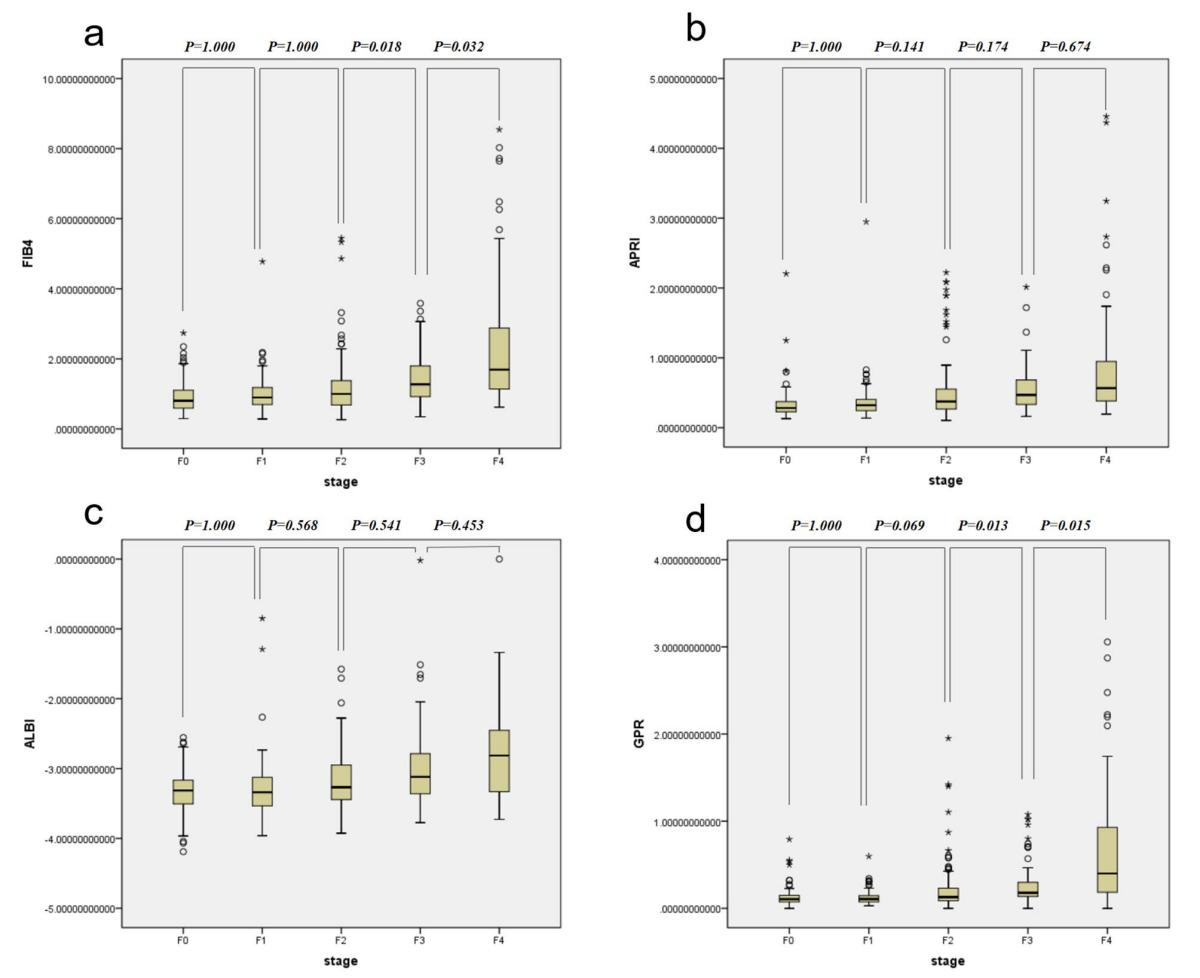
The diagnostic ability of the four fibrosis indices, FIB4, APRI, ALBI and GPR in differentiating liver fibrosis stage were evaluated. **(a)** FIB4 was able to distinguish stage 3 from 2 and stage 4 from 3(*P* < 0.05). **(b) **and **(c)** APRI and ALBI failed to distinguish stage 3 from 2 or stage 4 from 3 (*P* > 0.05). **(d)** GPR was able to distinguish stage 3 from 2 and stage 4 from 3(*P* < 0.05). *P*-values < 0.05 were considered statistically significant

FIB4, APRI, ALBI, AAR and GPR are known to be good predictors of cirrhosis and fibrosis. Median (interquartile spacing) was used to demonstrate their values in 5 groups, and FIB4, APRI, ALBI and GPR scores were significantly associated with liver fibrosis in our cohort (Table 3). Further, pairwise comparison of meaningful indicators between levels were carried on. We found FIB4 and GPR to be significantly associated with liver fibrosis in fully adjusted models, taking into account multiple testing correction (Fig. 2): FIB4 showed statistical differences between F2 and F3 group (*p* = 0.018) or F3 and F4 group (*p* = 0.032), respectively; GPR showed statistical differences between F2 and F3 group (*p* = 0.013) or F3 and F4 group (*p* = 0.015), respectively.

**Table 3:** Comparison of the FIB4, APRI, ALBI, AAR and GPR of the 433 patients in each stage of fibrosis

Fibrosis staging	F0	F1	F2	F3	F4	*P*
Patient number	93	83	111	75	71	
FIB4	0.81(0.52)	0.90(0.50)	1(0.71)	1.27(0.91)	1.71(1.82)	< 0.001
APRI	0.28(0.15)	0.32(0.18)	0.37(0.30)	0.47(0.36)	0.59(0.85)	< 0.001
ALBI	-3.31(0.34)	-3.34(0.42)	-3.27(0.50)	-3.12(0.59)	-2.81(0.95)	< 0.001
AAR	0.81(0.42)	0.82(0.40)	0.87(0.52)	0.88(0.40)	0.96(0.41)	0.062
GPR	0.11(0.08)	0.11(0.07)	0.13(0.15)	0.18(0.18)	0.40(0.75)	< 0.001

Continuous variables are presented as the median and interquartile range

### Diagnostic ability of distinguishing advanced fibrosis (F3-4) from non-advanced fibrosis (F1-2)

**Fig. 3:**
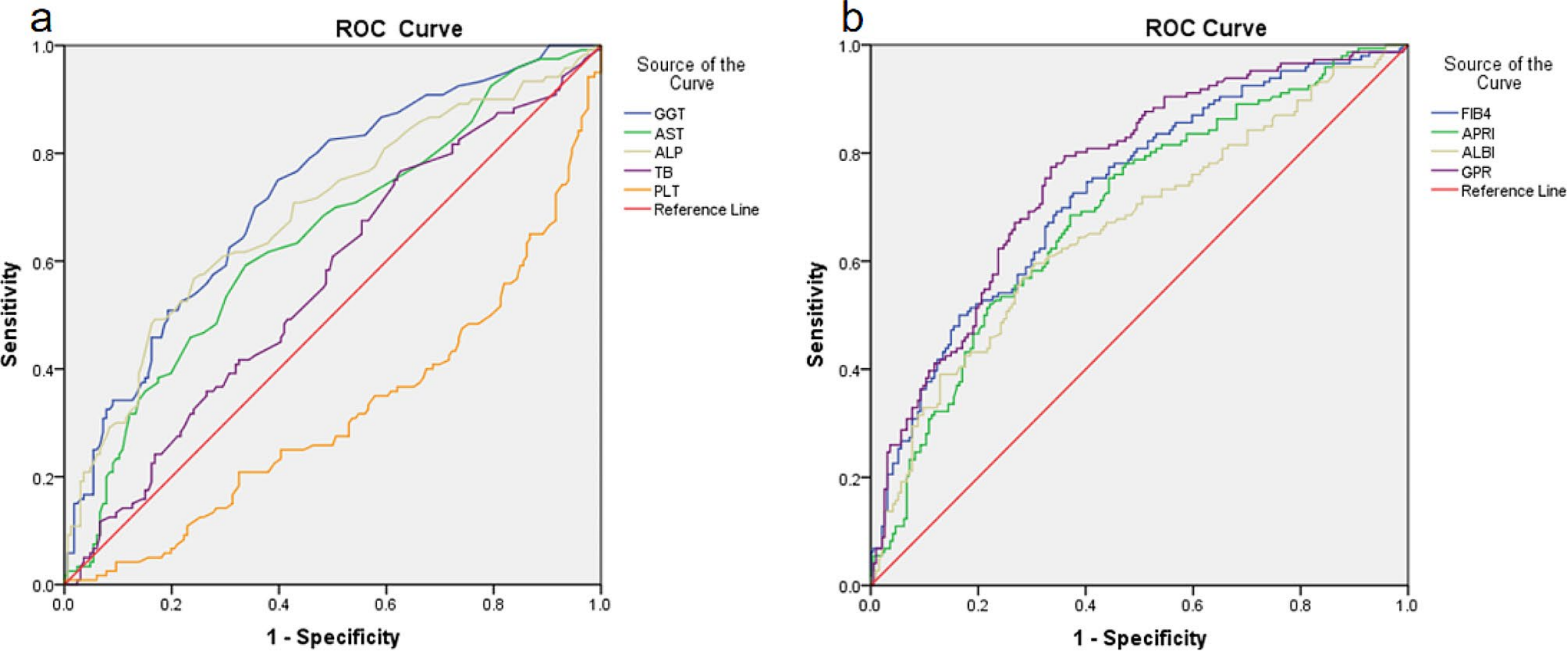
Receiver operating characteristic (ROC) analysis of non-invasive serum biomarkers of liver fibrosis indices to distinguish advanced fibrosis (F3-4) from non-advanced fibrosis (F1-2).** (a)** The AUROCs of the GGT was 0.723 (95%CI 0.668–0.777), a sensitivity of 73.6% and specificity of 62.0% indicated that GGT was able to distinguish advanced fibrosis (F3–4) from non-advanced fibrosis (F1-2). **(b)** The AUROCs of the FIB4 and GPR was 0.729 (95%CI 0.675–0.782) and 0.760 (95%CI 0.709–0.811), respectively, indicating the ability to distinguish advanced fibrosis (F3–4) from non-advanced fibrosis (F1–2)

Multivariate analysis was performed using a logistic regression model to discover the biomarker potential that could distinguish advanced fibrosis (F3-4) from non-advanced fibrosis (F1-2) among individuals with chronic hepatitis B, and then perform Receiver operating characteristic to find the cut-off point. GGT, FIB4 and GPR presented a better sensitivity and specificity than the other characters with AUROCs above of 0.70. The AUROCs of the GGT was 0.723 (95%CI 0.668–0.777), a sensitivity of 73.6% and specificity of 62.0%. The AUROCs of the FIB4 was 0.729 (95%CI 0.675–0.782), a sensitivity of 72.6% and specificity of 62.4%. The AUROCs of the GPR was 0.760 (95%CI 0.709–0.811), a sensitivity of 77.4% and specificity of 66.5%. The cut-off values of GGT, FIB4 and GPR for diagnose advanced fibrosis were 27.50, 1.075 and 0.147, respectively. Therefore, GPR was a better biomarker to identify advanced fibrosis (F3-4) and non-advanced fibrosis (F1-2) in patients with chronic hepatitis B(Table 4; Fig. 3).

**Table 4:** Multivariate analysis was performed to discover the biomarker potential that could distinguish advanced fibrosis (F3-4) from non-advanced fibrosis (F1-2)

	AUC	95%CI		CUT-POINT	Sensitivity	Specificity
ALP	0.689	0.626	0.752	76.50	0.567	0.674
AST	0.623	0.563	0.683	31.50	0.541	0.686
GGT	0.723	0.668	0.777	27.50	0.736	0.620
TB	0.551	0.489	0.613	9.550	0.740	0.376
PLT	0.670	0.612	0.728	159.000	0.804	0.466
FIB4	0.729	0.675	0.782	1.075	0.726	0.624
APRI	0.692	0.636	0.749	0.393	0.685	0.629
ALBI	0.663	0.604	0.722	-3.134	0.596	0.696
GPR	0.760	0.709	0.811	0.147	0.774	0.665

### Diagnostic ability of distinguishing cirrhosis (F4) from non-cirrhotic stages (F1-3)

**Fig. 4:**
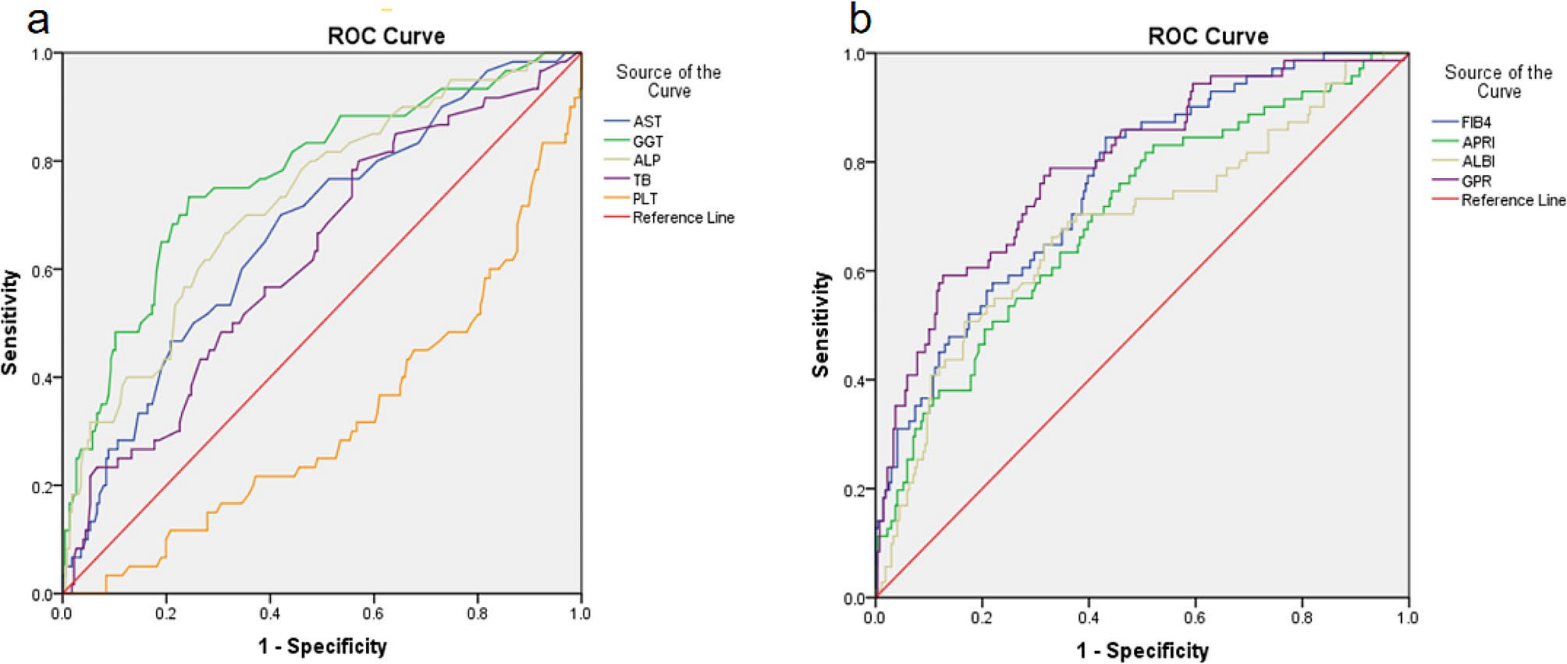
Receiver operating characteristic (ROC) analysis of non-invasive serum biomarkers of liver fibrosis indices to distinguish cirrhosis (F4) from non-cirrhotic stages (F1-3) **(a) **The AUROCs of the GGT was 0.775 (95%CI 0.711–0.840), a sensitivity of 71.4% and specificity of 76.7% with the cut-off values were 38.50, indicated that GGT was able to distinguish cirrhosis (F4) from non-cirrhotic stages (F1-3). **(b)** The AUROCs of the GPR was 0.794 (95%CI 0.734–0.853), but it had a lower sensitivity of 59.2%

Meanwhile, multivariate analysis showed that GGT was able to distinguish cirrhosis (F4) from non-cirrhotic stages (F1-3) with AUROCs of 0.775 (95%CI 0.711–0.840), a sensitivity of 71.4% and specificity of 76.7%, with the cut-off values were 38.50. The AUROCs of the GPR was 0.794 (95%CI 0.734–0.853), but it had a lower sensitivity of 59.2% (Table 5; Fig. 4). Therefore, GGT was a better biomarker to identify cirrhosis (F4) from non-cirrhotic stages (F1-3) in patients with chronic hepatitis B. However, none of the above biomarkers presented a better sensitivity and specificity to distinguish liver fibrosis stages (F2-4) from hepatitis phase (F0-1).

**Table 5:** Multivariate analysis was performed to discover the biomarker potential that could distinguish cirrhosis (F4) from non-cirrhotic stages (F1-3)

	AUC	95%CI		CUT-POINT	Sensitivity	Specificity
ALP	0.726	0.654	0.798	75.50	0.667	0.686
AST	0.662	0.591	0.732	30.50	0.662	0.602
GGT	0.775	0.711	0.840	38.50	0.714	0.767
TB	0.617	0.544	0.689	10.05	0.803	0.439
PLT	0.663	0.591	0.736	154.50	0.770	0.507
FIB4	0.761	0.701	0.821	1.08	0.845	0.569
APRI	0.700	0.631	0.770	0.37	0.817	0.494
ALBI	0.682	0.607	0.757	-2.82	0.507	0.833
GPR	0.794	0.734	0.853	0.33	0.592	0.874

## Discussion

Early diagnosis of the stage of liver fibrosis is essential to control the progression of hepatitis B infection. According to the stage of liver fibrosis, timely clinical intervention measures can prevent further progression to advanced liver fibrosis or hepatocellular carcinoma [12, 19]. Liver biopsy is still the gold standard for assessing the staging of liver fibrosis, but it is an invasive procedure that limits its widespread application in clinical practice [20]. Studies have shown that several serum biomarkers, such as GPR, AAR, APRI, ALBI and FIB-4 can distinguish advanced fibrosis from non-advanced fibrosis [7–9, 21]. The accurate staging of liver fibrosis by these biomarkers will reduce or replace liver biopsy for liver fibrosis staging [10, 22]. However, the majority of studies have focused on patients with hepatitis C, and there are few studies on the ability of biomarkers to stage liver fibrosis in patients with hepatitis B infection. The present study yielded the following results. First, the proportion of males with progressive stages of liver fibrosis was relatively larger, and the average age of patients with cirrhosis stages is older than the non-cirrhotic stages. Second, We found PLT, GGT, ALP, TB, FIB4 and GPR to be significantly associated with liver fibrosis in our cohort. GGT, GPR was able to distinguish cirrhosis (F4) from non-cirrhotic stages (F1-3), while GGT, FIB4 and GPR could distinguish advanced fibrosis (F3-4) from non-advanced fibrosis (F1-2) among individuals with chronic hepatitis B. Finally, Among these biomarkers, GGT was a better biomarker to distinguish cirrhosis (F4) from non-cirrhotic stages (F1-3), while GGT was a better biomarker to identify cirrhosis (F4) from non-cirrhotic stages (F1-3) in patients with chronic hepatitis B.

In this study, the proportion of males with progressive stages of liver fibrosis was relatively larger, and the average age of patients with cirrhosis stages is older than the non-cirrhotic stages. This conclusion is consistent with the results of previous studies. In patients with compensated cirrhosis, the proportion of asymptomatic patients is 30-40%, and they are often found only during physical examination, surgery, or even autopsy. Studies have shown that the number of cases of liver cirrhosis increased by 74.5% worldwide from 1990 to 2017, of which non-alcoholic fatty liver disease (NAFLD) accounted for 59.5%, and chronic hepatitis B (CHB) accounted for 28.7% [23]. An epidemiological study in 2020 showed that the mortality rate of chronic liver disease was 12.86 per 100,000 people and the mortality rate of cirrhosis was 7.96 per 100,000 people in the United States from 1999 to 2017 [24]. From 1990 to 2016, the number of patients with liver cirrhosis and chronic liver disease in China increased from nearly 7 million to nearly 12 million, and the prevalence and mortality of males were higher than those of females [25]. The disease progression rate of viral hepatisis-related liver fibrosis, cirrhosis and HCC is related to host factors such as age, gender, ethanol intake, intrahepatic fat deposition and insulin resistance [26]. These factors, especially age and sex, have been strongly demonstrated in model studies of liver disease progression [27]. The age of HCV infection is significantly related to the risk of disease and the progression rate of liver fibrosis. The disease progression rate is faster in patients over 50 years old, and the disease progression rate is faster in young men than in women [28, 29].

We found GGT was able to distinguish cirrhosis (F4) from non-cirrhotic stages (F1-3), and advanced fibrosis (F3-4) from non-advanced fibrosis (F1-2) among individuals with chronic hepatitis B. Previous studies have demonstrated GGT is important predictors of significant fibrosis or cirrhosis. Splenoportal index (SPI) and liver fibrosis index (LFI) were found to be independent predictors of significant fibrosis, whereas GGT, SPI and LFI were independent predictors of cirrhosis [30]. Lens et al. found that based on multivariate analysis, the only variables identified as independent predictors of cirrhosis were age, fibrosis stage, GGT and AST at baseline [31] Imbert-Bismut et al. found that a combination of five or six basic biochemical markers can have high positive or negative predictive value for diagnosis of clinically significant fibrosis, even at the early stage of a few septa, which could be used to substantially reduce the number of liver biopsies done in patients. The most informative markers were, in decreasing rank: a2 macroglobulin, haptoglobin, GGT, globulin, total bilirubin, and apolipoprotein A1 [32]. GGT and bilirubin were both associated with hepatocyte growth factor, which is a pleiotropic cytokine produced by hepatic stellate cells. Early cholestasis or an increase of epidermal growth factor could be one explanation for the observed increase in GGT with increasing fibrosis severity [33].

In our study, we found FIB4 could distinguish advanced fibrosis (F3-4) from non-advanced fibrosis (F1-2) among individuals with chronic hepatitis B. As reported by Vallet-Pichard et al. [34], the FIB-4 index, a simple, accurate, and inexpensive method, enabled the correct identification of patients with severe fibrosis (F3-F4) and cirrhosis with an area under the receiver operating characteristic curve of 0.85 (95%CI 0.82–0.89) and 0.91 (95%CI 0.86–0.93), respectively. An FIB-4 index < 1.45 had a negative predictive value of 94.7% to exclude severe fibrosis with a sensitivity of 74.3%. An FIB-4 index higher than 3.25 had a positive predictive value to confirm the existence of a significant fibrosis (F3-F4) of 82.1% with a specificity of 98.2%. Kim et al. [35]. found that FIB-4 is a simple, accurate and inexpensive method for prediction of significant (F ≥ 2) and severe (F ≥ 3) fibrosis, and cirrhosis (F = 4), the area under the receiver-operating characteristic curves were 0.865, 0.910 and 0.926 respectively. However, Huang et al. [12]. foud that though the FIB-4 were significantly different between stages of LF, no significant differences in the FIB-4 were found between stage F0 and F1 or between stage F3 and F4. This may be related to the fact that FIB-4 may be affected by many factors such as age and the degree of liver inflammation. FIB-4 is simple and easy to calculate at the bedside or in an outpatient clinic, and can make 70.5% of patients avoid liver biopsy [35].

GPR could distinguish advanced fibrosis (F3-4) from non-advanced fibrosis (F1-2) among individuals with chronic hepatitis B. With the METAVIR liver pathological scoring system as reference, the preliminary investigation indicated that the performance of GPR in predicting significant fibrosis (≥ F2), extensive fibrosis (≥ F3) and cirrhosis (≥ F4) was close to or higher than those of APRI and FIB-4 [36]. Lemoine et al. found that the GPR is a more accurate routine laboratory marker than APRI and FIB-4 to stage liver fibrosis in patients with CHB in West Africa, which was useful in predicting the levels of liver fibrosis of CHB patients [37]. However, Li et al. found that GPR does not show advantages than APRI and FIB-4 in identifying significant fibrosis, severe fibrosis, and cirrhosis in CHB patients in China [38]. Difference between performances may be related to difference in disease phenotype and HBV genotype between heterogeneous populations. This suggest the GPR deserves to be further validated in different populations.

There are some shortcomings in our study. First, our patients were enrolled from a single referral center, which can lead to selection bias.Besides, this is a single-center study with a limited number of patients and lacks a validation component, so further studies with a larger sample size from multiple centers are needed to validate the generalizability and robustness of our identified biomarkers for liver fibrosis staging in HBV infection. Third, our findings may not be applicable in other countries and regions, further research conducted among other populations are warranted to provide more evidence.

In conclusion, the results of this study show that PLT, GGT, ALP, TB, FIB4 and GPR were significantly associated with liver fibrosis in our cohort. GGT and GPR was able to distinguish cirrhosis (F4) from non-cirrhotic stages (F1-3), while GGT, FIB4 and GPR could distinguish advanced fibrosis (F3-4) from non-advanced fibrosis (F1-2). Among these biomarkers, GGT was a better biomarker to distinguish cirrhosis (F4) from non-cirrhotic stages (F1-3), while GPR was a better biomarker to identify advanced fibrosis (F3-4) and non-advanced fibrosis (F1-2) in patients with chronic hepatitis B.

### Electronic supplementary material

Below is the link to the electronic supplementary material.

Supplementary Material 1

## Data Availability

Data is provided within the manuscript.
